# Using photogrammetry to create virtual permanent plots in rare and threatened plant communities

**DOI:** 10.1002/aps3.11534

**Published:** 2023-08-18

**Authors:** Andrea J. Tirrell, Aaron E. Putnam, Michael I. J. Cianchette, Jacquelyn L. Gill

**Affiliations:** ^1^ School of Biology and Ecology University of Maine Orono Maine 04469 USA; ^2^ Climate Change Institute University of Maine Orono Maine 04469 USA; ^3^ School of Earth and Climate Sciences University of Maine Orono Maine 04469 USA

**Keywords:** alpine plant communities, digital surface model (DSM), long‐term monitoring, photogrammetry, plant conservation, rare plant communities

## Abstract

**Premise:**

Many plant communities across the world are undergoing changes due to climate change, human disturbance, and other threats. These community‐level changes are often tracked with the use of permanent vegetative plots, but this approach is not always feasible. As an alternative, we propose using photogrammetry, specifically photograph‐based digital surface models (DSMs) developed using structure‐from‐motion, to establish virtual permanent plots in plant communities where the use of permanent structures may not be possible.

**Methods:**

In 2021 and 2022, we took iPhone photographs to record species presence in 1‐m^2^ plots distributed across alpine communities in the northeastern United States. We then compared field estimates of percent coverage with coverage estimated using DSMs.

**Results:**

Digital surface models can provide effective, minimally invasive, and permanent records of plant species presence and percent coverage, while also allowing managers to mark survey locations virtually for long‐term monitoring. We found that percent coverage estimated from DSMs did not differ from field estimates for most species and substrates.

**Discussion:**

In order to continue surveying efforts in areas where permanent structures or other surveying methods are not feasible, photogrammetry and structure‐from‐motion methods can provide a low‐cost approach that allows agencies to accurately survey and record sensitive plant communities through time.

As some of Earth's most vulnerable plant communities face growing threats due to global change (Walther et al., 2002; Mutke and Barthlott, 2005; Körner et al., 2011), long‐term monitoring remains a critical tool for understanding and tracking species responses (Fancy and Bennetts, 2012; Elmendorf et al., 2015; Bjorkman et al., 2020; Ravolainen et al., 2020). Mountain ecosystems are particularly threatened, due to the impacts of climate change (Tinner and Ammann, 2005; Steinbauer et al., 2018; Harrison, 2020; Nomoto and Alexander, 2021) and human‐mediated disturbance such as development and increasing hiker visitation (Barros et al., 2020; Rashid et al., 2021). However, the response of alpine and tundra plant communities to these changes may vary across regions (Walker et al., 2006), and previous studies have predicted both increases and decreases in plant species richness and diversity depending on factors including geographic location, species interactions, and biogeochemical cycling (Walker et al., 2006; Steinbauer et al., 2018; Harrison, 2020). Together, these factors highlight the importance of localized monitoring to track the response of sensitive or rare plant communities to complex global changes (Elmendorf et al., 2015). However, despite the well‐recognized importance of long‐term monitoring for understanding plant community change through time, field methods such as permanent plots can be detrimental to the very communities being studied. Trampling by hikers (or researchers) has been found to impact overall vegetation cover (Cole, 1995), as well as soil microbial communities, while increasing soil erosion (Liddle, 1975; Morgan and Smith, 1980; Lucas‐Borja et al., 2011). While some level of trampling is unavoidable during fieldwork, minimizing the time spent in the field could help reduce damage to vulnerable plant communities, which are already facing threats from growing numbers of recreational visitors (Barros et al., 2020).

Long‐term monitoring methods have been established for many rare and threatened plant communities across the globe. For instance, the Global Observation Research Initiative in Alpine Environments (GLORIA; https://www.gloria.ac.at/) seeks to understand how alpine plant communities are responding to climate change through the use of standardized surveys (Pauli et al., 2015). GLORIA has demonstrated the importance of long‐term monitoring in alpine environments by validating elevational shifts in species distributions through time (Pauli et al., 2005; Grytnes et al., 2014), as well as changes in alpine plant diversity (Gigauri et al., 2014; Lamprecht et al., 2021; Steinmann et al., 2021) and shifts in phenology (Pelayo et al., 2021). The GLORIA protocol requires target regions to have a minimum of four summits, with all summits having consistent local climates and bedrock types, and not being heavily affected by human activity (Pauli et al., 2015). Including summits that are heavily trafficked by tourists (e.g., by hiking or ski resorts) is discouraged (Pauli et al., 2015). Therefore, while the GLORIA protocol is useful for many summits in regions where alpine areas are plentiful, remote, and relatively undisturbed (e.g., the Rocky Mountains of western North America, the Andes of South America, or the Alps of central Europe), many other alpine areas are too small, too isolated, or too heavily visited to qualify, despite being ecologically important (e.g., the tundra relicts on the summits of many peaks in the northeastern United States). GLORIA protocols also require the installation of permanent structures with aboveground and belowground components, such as aluminum tubing, to mark plot locations so they can be revisited (Pauli et al., 2015). In the case of rare alpine plant communities, the installation of permanent structures above treeline is strictly prohibited by most federal and state agencies in the northeastern United States, as such structures can damage already thin and easily disturbed alpine soils (Poulenard and Podwojewski, 2006), making their use more harmful than beneficial.

Rare or threatened plant communities are often managed by small and diffuse organizations, which presents another challenge to long‐term monitoring. Alpine areas of the northeastern United States thus serve as a case study of systems where traditional long‐term monitoring approaches are challenging. These insular alpine ecosystems are managed by a myriad of state, federal, and private agencies, each with their own rules and guidelines for scientific research. Most agencies in this region do not permit the establishment of permanent structures in the alpine zone, whether due to the possibility of hikers being lured off‐trail, the disturbance of alpine soils, or interfering with the wilderness experience for park visitors (Mount Mansfield Science and Stewardship Center, 2016; Baxter State Park, 2023). All of the factors that exclude most northeastern U.S. alpine communities from GLORIA monitoring are the same factors that make these areas especially vulnerable and in need of continued, standardized monitoring, including high visitation rates, development, and climate change. New methods for long‐term monitoring, such as the use of structure‐from‐motion photogrammetry (specifically digital surface models, or DSMs), can be helpful in ecosystems where traditional monitoring protocols are not useful.

## Low‐cost photogrammetry for applied conservation efforts

The use of orthorectified imagery can serve as a solution to problems with long‐term monitoring of rare and threatened plant communities. Photogrammetry is defined as the use of photographic images to record and measure objects (Ferrari et al., 2021), often by converting a series of overlapping two‐dimensional (2D) images into three‐dimensional (3D) models. In structure‐from‐motion photogrammetry, a photographer (or oftentimes, an unmanned aerial vehicle [UAV]) captures multiple overlapping photos in a 360° coverage around a target object, including ground‐level, chest‐level, and bird's eye view perspectives. During post‐processing, these photos are cross‐referenced and aligned within photogrammetric software, generating a 3D point‐based representation of the target landscape. This software takes advantage of parallax relationships to create multiple kinds of digital 3D models (like DSMs), which consist of a mosaic of images that are corrected for geometric errors or distortions (Ferrari et al., 2021). These models can then be georeferenced with GPS or differential GPS (DGPS) coordinates, effectively grounding the model in geographic space. Photogrammetric models have been used in many disciplines, from mapping Earth surface features, such as glacial and fluvial landforms (Lane et al., 1993; Marteau et al., 2017; Mayewski et al., 2020; Strand et al., 2022), to aerial monitoring of forest trees (Krause et al., 2019; Zhang et al., 2019), to undersea monitoring and restoration of coral reef ecosystems (Calders et al., 2020; Ferrari et al., 2021), but are as‐yet underutilized in the monitoring of rare plant communities.

Often, images included in the photogrammetric model (i.e., DSMs) are captured using advanced and remote technologies such as light detection and ranging (LiDAR) or synthetic aperture radar devices mounted on UAVs (Valbuena et al., 2020). These tools allow investigators to sample large areas of interest while recording highly detailed data about plant communities, ecosystem properties, functional traits, and geospatial data (Ferrari et al., 2021). Such technologies allow for the acquisition of high volumes of biodiversity data (Valbuena et al., 2020); however, they are often costly to implement, which can make them inaccessible for some agencies or researchers. By removing cost barriers associated with equipment, photogrammetry can remain a useful tool for long‐term monitoring, even with a limited budget.

**Figure 1:**
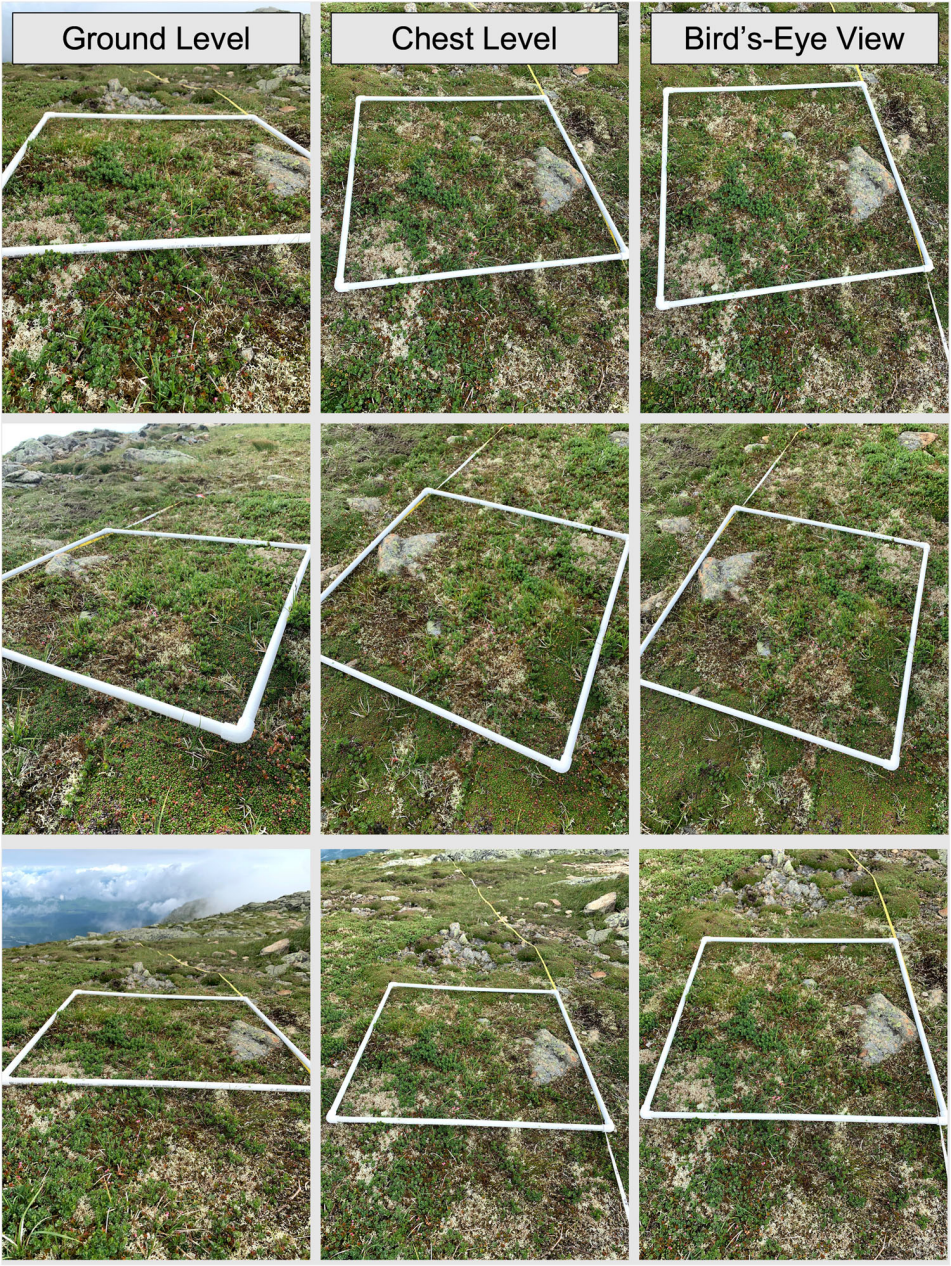
A selection of photos taken with an iPhone 10, used to create a digital surface model (DSM) or virtual permanent plot on Mt. Lafayette, New Hampshire, USA. Between 30 and 50 overlapping images taken at ground level, eye level, and bird's eye view of a quadrat can be incorporated into a DSM using photogrammetric software with enough resolution to identify species and estimate plant or substrate coverage.

Here, we present an approach to photogrammetry that leverages the use of accessible technology to create “virtual” permanent plots for the long‐term monitoring of vulnerable plant communities, such as in alpine ecosystems where traditional monitoring approaches may not be feasible. When more sophisticated imaging technology is not available, the images needed for DSMs can be collected by hand using widely available digital cameras, including smartphones (Figure 1). Because DSMs can be created using equipment that is (1) readily available, (2) low‐cost, and (3) lightweight, photogrammetry may be especially well‐suited for use by small agencies in remote areas (including volunteers or citizen scientists), or in locations where UAVs or other tools are cost‐prohibitive, unfeasible, or not permitted. To test the utility of this approach, in 2021 and 2022, we completed alpine plant survey transects across eight mountains in the northeastern United States and photographed two to three random plots per site using an iPhone 10 (Apple, Cupertino, California, USA). These photos were then used to create georeferenced DSMs using Agisoft Metashape version 1.8 (Agisoft, St. Petersburg, Russia; https://www.agisoft.com/). Our DSMs serve as an accurate record of plant species coverage and presence at a particular time. Our DSMs can also be used to easily find plot locations in the field so they can be rephotographed by future surveyors.

## METHODS

During the summers of 2021 and 2022, we completed plant surveys at eight alpine field sites across Vermont, New Hampshire, and Maine, USA. We deployed 1‐m^2^ quadrats at each contiguous meter along 50‐m transects and identified all vascular plant species present, as well as estimated the percent coverage of all plants and substrates. During each survey, we used a random number generator to select two to three quadrats to photograph for the incorporation in photogrammetric models. Each photographed quadrat was also marked with GPS or DGPS coordinates, using either a Garmin In‐Reach (Garmin, Schaffhausen, Switzerland) or an Emlid RS2+ (Emlid, Budapest, Hungary). Overlapping photographs were taken at ground level, chest level, and from bird's eye view at each corner and along the side of the quadrat with an iPhone 10 camera (Figure 1). Multiple overlapping photos were needed to cover each side of the quadrat horizontally (2–4 photos per side, depending on time available for photographing). Between 30 and 50 overlapping images are required for each 1‐m^2^ quadrat to provide the imaging software with sufficient information to generate an accurate 3D model. Each site took approximately 10–20 minutes to photograph. We also “virtually” marked the beginning of each transect in the field using this method, to provide a record of the transect locations for future repeat surveys.

**Figure 2:**
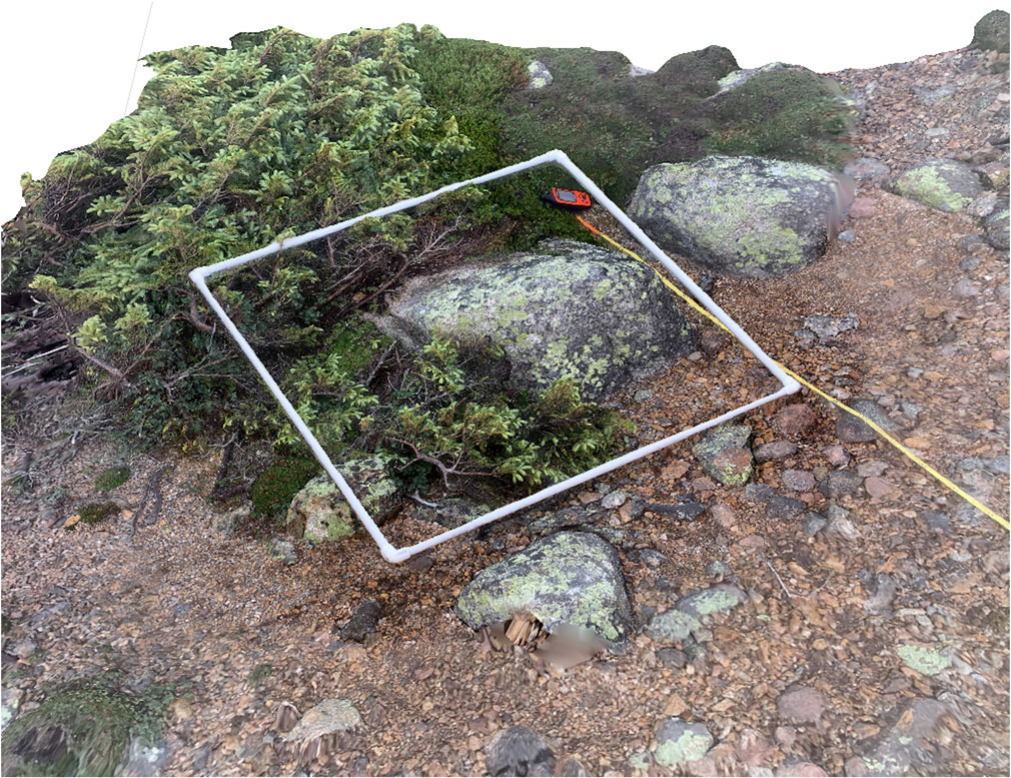
A completed digital surface model of a 1‐m^2^ quadrat on Mt. Guyot, New Hampshire, USA (rendered here in two dimensions). This model was rendered from 40 photos taken with an iPhone 10.

Field photos were converted to DSMs using Agisoft Metashape and then georeferenced with QGIS software (QGIS Geographic Information System, Version 3.18.0‐Zürich, http://www.qgis.org; Figure 2, Video S1). To create DSMs in Agisoft Metashape software, we: (1) uploaded the photos as JPEGs, (2) aligned the photos in space, (3) built a polygonal mesh from the point cloud, and (4) built the texture, which adds finer details to the model (Table 1; Agisoft Helpdesk Portal, 2022). The model was then saved as a PSX file and exported as an OBJ file, as well as a 2D orthomosaic (but can also be exported as other file formats, including TIFF and JPEG). For steps 1 and 2, the software then integrated the photos, estimating the camera position to render 2D images into a 3D point cloud. The point cloud was then cropped using the “selection” feature, which eliminates anything the user does not want to include in the image (e.g., people, objects, background noise). During steps 3 and 4, the software algorithm constructed the mesh geometry and textural detail of the model (Agisoft Helpdesk Portal, 2022). After georeferencing the models using GPS‐determined ground‐control points (GCPs), the DSMs can be viewed in free apps, such as Agisoft Metashape Viewer and Google Earth Pro, or other mapping software packages. DSMs and associated images can later be brought into the field and, with differential GPS or geotagged coordinates from smartphones, used to relocate survey plots for subsequent monitoring through new DSMs or field sampling. Through this process, we were able to survey and permanently record species presence and coverage across eight alpine zones with minimal disturbance and no permanent structures.

**Table 1:** Suggested workflow for creating a digital surface model in Agisoft Metashape software (Agisoft Helpdesk Portal, 2022).

Model creation workflow	Actions	Dialog box settings
Workflow → Add photos	Select all relevant photos to be included in the DSM. Ensure that photos are in the correct format before adding photos (i.e., JPEG, PNG).	
Workflow → Align photos	After photos are aligned, delete unnecessary tie points by selecting and removing points outside of the bounding box.	Accuracy → High ✓Generic preselection
Workflow → Build mesh	Choosing high‐quality mesh building will require considerable computing power; consider choosing medium quality to save time.	Source data → Depth maps Surface type → Arbitrary (3D) Quality → Medium or High Face count → High Depth filtering → Mild ✓Calculate vertex colors
Workflow → Build texture Right click → Add marker	Save the DSM as an Agisoft Metashape file (.psx) after building texture. This ensures the model can be edited again if needed. At this step, a scale bar can be created by measuring the distance between two markers (or points) within the DSM and then selecting “Create Scale Bar.”	Texture type → Diffuse map Source data → Images Mapping mode → Generic ✓Enable hole filling ✓Enable ghosting filter
Workflow → Export model	At this step, the model can be converted to a JPEG, TIFF, etc.	

To evaluate the accuracy of plant coverage characterizations using the photogrammetric approach, we compared percent coverage values estimated in the field with coverage values estimated from our DSMs in the lab, three months after fieldwork was completed. A field assistant, who was familiar with the species present at our field sites, was provided with eight random DSMs from eight different sites but was not provided with field site or transect location to minimize bias. Using only the DSM, the assistant visually estimated percent coverage just as they would in the field. This included the estimation of all vascular plant species present, as well as substrates like rock and gravel. DSMs were scaled in Agisoft Metashape by manually creating scale bars, utilizing the known length of each side of the quadrat. The assistant also estimated coverage of all species and substrates in 2D bird's eye view images that were incorporated into the same DSMs as above. Two‐dimensional images also included scale bars, created with the known length of quadrat sides.

To test whether estimates of percent coverage from the field and virtual quadrats were similar, we used linear regression models and root‐mean‐square error or deviation (RMSE). Linear regression models of field estimates and DSM estimates were used to analyze differences in percent coverage on the bulk plant data and substrate (all species and substrates lumped together) or by individual species or substrate. Field‐collected estimations served as the explanatory variable, while DSM‐collected estimations served as the response variable. RMSE (in the Metrics package in R) values were quantified to measure the accuracy of the models at predicting percent coverage (Hamner and Frasco, 2018; R Core Team, 2022). High RMSE values indicate poor model fit, or dissimilarity between field‐estimated and DSM‐estimated coverage, while lower values indicate a better model fit, or similarity between field‐estimated and DSM‐estimated coverage. These methods were then used to evaluate the difference between field‐estimated and 2D image–estimated percent coverage, in order to evaluate how 3D image estimation might resemble or differ from 2D point‐image estimations.

## RESULTS

**Figure 3:**
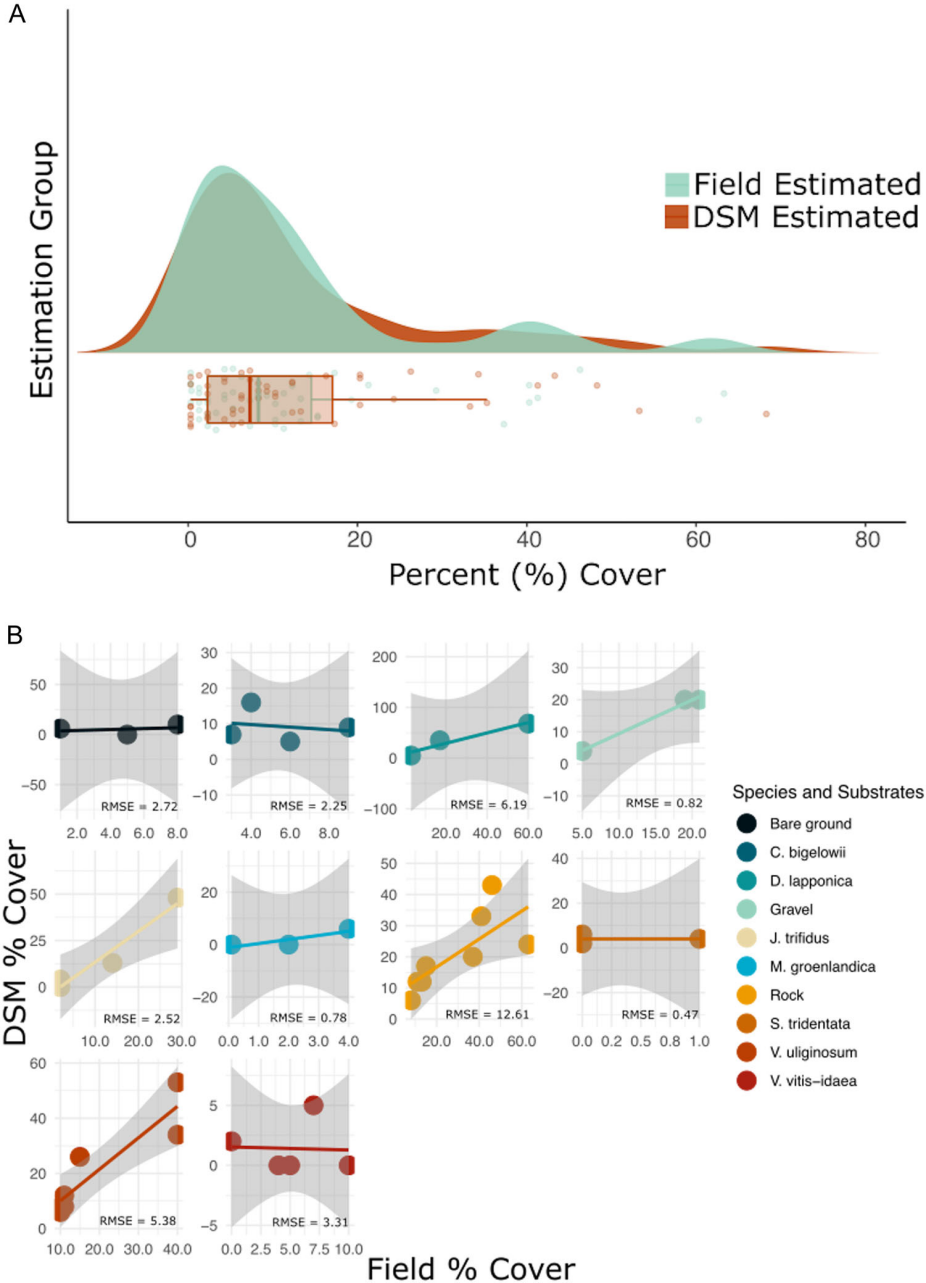
Results from comparing percent coverage from field estimations, 2D image estimations, and DSM estimations. (A) A raincloud plot (with density plot above and raw data points below, plus box‐and‐whisker plots showing the first to third quantiles) illustrating the overlap between field‐estimated percent coverage values and coverage estimated from DSMs. (B) Scatterplots showing DSM‐estimated coverage against field‐estimated coverage, where each facet represents a different species or substrate, with associated trendlines and 95% confidence intervals. Root‐mean‐square deviation (RMSE) values are shown in the bottom right corner of each plot. Both the *x*‐ and *y*‐axes differ across species to allow for visualization.

Results from our first linear regression, which includes all species and substrates estimations, revealed significant differences between field‐estimated and DSM‐estimated values (*P* < 0.01, RMSE = 8.65), which was an unexpected outcome. However, when species and substrate are accounted for in the models, results indicate that most species are very well estimated in DSMs compared to field estimates (Figure 3A). This suggests that a handful of species and/or substrates control the linear regression when all are binned together. Species and substrates where DSM estimates and field estimates were essentially equivalent included: bare ground (*P* = 0.80, RMSE = 2.72), gravel (*P* = 0.07, RMSE = 0.82), *Carex bigelowii* Torr. ex Schwein. (*P* = 0.80, RMSE = 2.25), *Minuartia groenlandica* (Retz.) Ostenf. (*P* = 0.32, RMSE = 0.78), *Sibbaldiopsis tridentata* (Aiton) Rydb. (*P* = 1.00, RMSE = 0.47), *Diapensia lapponica* L. (*P* = 0.13, RMSE = 6.19), and *Vaccinium vitis‐idaea* L. (*P* = 0.94, RMSE = 3.31; Figure 3B). Species and substrates that were overestimated in the DSMs as compared to the field estimations included: rock (*P* = 0.03, RMSE = 12.61), *Juncus trifidus* L. (*P* = 0.02, RMSE = 2.50), and *Vaccinium uliginosum* L. (*P* < 0.01, RSME = 5.40; Figure 3B). Species and substrates for which we did not have sufficient data included *Abies balsamea* (L.) Mill., *Betula papyrifera* Marshall, *Empetrum nigrum* L., *Huperzia appressa* (Desv.) Á. Löve & D. Löve, *Picea* spp., *Rhododendron groenlandicum* (Oeder) Kron & Judd, *Rhododendron lapponicum* (L.) Wahlenb., and dead wood, likely because these species and substrates were less common across quadrats.

Linear regressions using 2D‐estimated percent coverage values yielded similar results. Two‐dimensional estimated values were significantly different than what was estimated in the field (*P* < 0.01, RSME = 7.04). However, when species and substrate were accounted for, 2D image estimations resembled our 3D estimations. Species and substrate estimations that were essentially equivalent to our field estimations included: bare ground (*P* = 0.38, RMSE = 1.60), gravel (*P* = 0.11, RMSE = 1.30), *Carex bigelowii* (*P* = 0.89, RMSE = 2.27), *Minuartia groenlandica* (*P* = 0.40, RMSE = 0.95), *Sibbaldiopsis tridentata* (*P* = 0.66, RMSE = 0.41), and *Vaccinium vitis‐idaea* (*P* = 0.90, RMSE = 1.90). Species and substrates that were overestimated in the 2D images included: rock (*P* = 0.09, RMSE = 14.7), *Diapensia lapponica* (*P* = 0.03, RMSE = 1.20), *Juncus trifidus* (*P* = 0.03, RMSE = 2.70), and *Vaccinium uliginosum* (*P* = 0.09, RMSE = 4.80).

## DISCUSSION

The creation of photogrammetric models from field photos has many advantages for the long‐term monitoring and conservation of rare and threatened plants. Not only is this process minimally destructive, avoiding disturbance to shallow soils and biocrusts that might result from emplacement of permanent structures (Poulenard and Podwojewski, 2006), but the creation of virtual permanent plots allows managers to maintain records of vascular plant coverage through time with minimal resources. Without non‐destructive means of surveying, some rare and threatened plant communities would be excluded from monitoring efforts such as GLORIA that require permanent structures and long hours in the field, among other requirements. The use of low‐cost photogrammetry methods can support rare plant community surveying efforts that do not fall under the purview of other long‐term monitoring methods and, as areas are resurveyed in the future, can provide conservation agencies with a record of plant presence through time.

**Figure 4:**
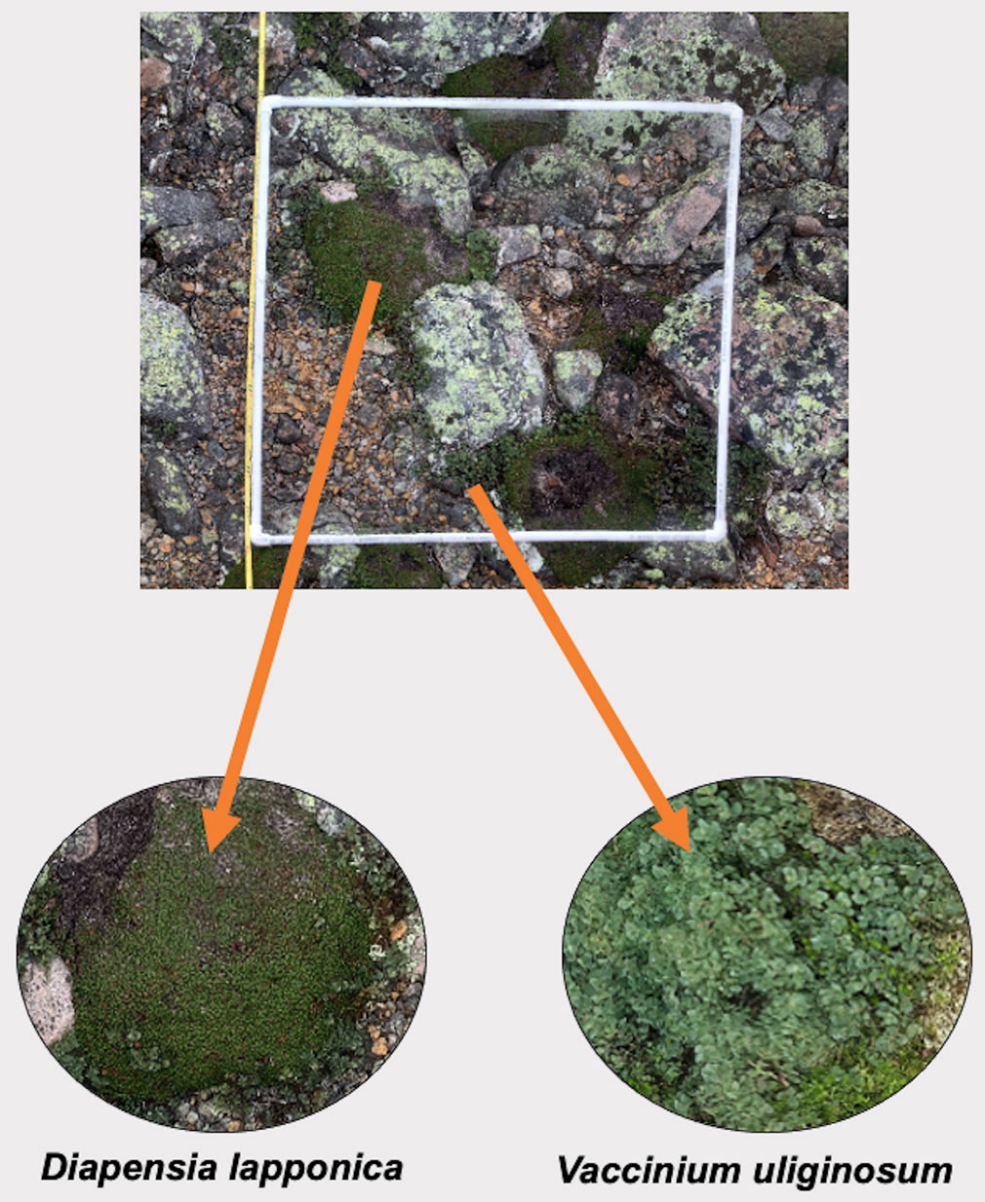
Vascular plants can be identified within digital surface models after fieldwork is complete in order to measure their coverage within a quadrat. Resolution within the DSM is high enough to allow identification of recognizable vascular plant species. Resolution is likely not high enough to enable identification of nonvascular plants, algae, or fungi unless more photos are incorporated.

Even while using low‐cost or low‐tech alternatives to UAVs for photogrammetry (e.g., common smartphones with geotagging features), the image quality of photogrammetric models is sufficient to identify and measure vascular plant species within a 1‐m^2^ quadrat (Figures 2, 4). Plant coverage values, which are normally estimated in the field, can also be estimated within DSMs, using both shape‐based measurements in Agisoft Metashape or visual estimation. Results from our analyses show that estimation using DSMs and 2D images are comparable to field‐estimated data. However, photogrammetric models, including DSMs, differ from singular point‐images in that they can be georeferenced and therefore are grounded in space and easily repeatable. Furthermore, photogrammetric models make plant identification easier and consistently accurate, as smaller, prostrate species were more often missed during 2D point‐image estimation. While 2D‐estimated coverage was comparable to field‐estimated values, the creation of photogrammetric models allows for the capturing of a wealth of data that cannot be captured in a 2D image, such as habitat complexity and functional trait data (Ferrari et al., 2021).

Overall, our method takes less time in the field compared to conventional estimation methods, which can be advantageous in locations where adverse conditions may disrupt fieldwork. Our photogrammetric approach can also provide inclusive opportunities for those who are unable to access field sites to participate in vegetation surveys. Photogrammetry thus allows investigators to record a large amount of community‐, population‐, and individual‐level data without spending extensive time in the field. It is also possible to measure some basic plant traits with 3D image models (Leménager et al., 2023), such as plant height using Agisoft Metashape measuring tools (which is not possible with singular point‐images, as they are not georeferenced). This advantage is especially important in the monitoring of alpine and Arctic plant communities, where changes in plant height are often a response to warming through time (Bjorkman et al., 2018). Photogrammetric models can also help with data reproducibility, repeat measurements, and can even be used in the classroom or in citizen science projects.

As with fieldwork, it is important that the image analyst has familiarity with the plant community at the field site to ensure accurate identification of the focal species after image processing. It should be noted that this need not be the same person who takes the images; our approach could also support fieldwork participation by those who cannot access the field site, such as remote collaborators or those with disabilities. Once species identification is completed (Figure 4), individual plants can be measured and percent coverage of species can be estimated either by traditional visual estimation or using Agisoft Metashape's measuring and drawing tools. It is important to note that the approach reported here may not be suitable for all taxa; for example, nonvascular plants, such as mosses, as well as lichens and fungi, would be challenging to identify using DSMs, as they often require the use of microscopes or reagents for identification even in the field. However, estimating percent coverage of mosses and lichens as a group is possible within the DSM, as it is with rock, bare ground, or other substrates. Small or infrequent species should be noted while in the field, as these species were most often missed during estimation in both DSMs and 2D point‐images.

In addition to their utility in reducing time and trampling in the field, the models produced by photogrammetry can also be reliably used to relocate field sites. One of our alpine field sites on Katahdin (Baxter State Park, Maine, USA) had been previously surveyed in 1989 by Hudson and Cogbill (1990); at the time, transect locations were recorded with film cameras, as well as by marking the undersides of rocks to indicate the starting point of each transect. In the field, however, we found it challenging to perceive depth or distance from the film photos (especially in cloudy or foggy conditions), which made it difficult to relocate these transect sites 33 years later by singular point‐images alone. Even with a 2D singular point‐image and GPS points, we found that it can be difficult to locate exact transect locations, both because GPS points taken using conventional handheld devices may include horizontal errors of several meters and because 2D point‐images are often missing important context clues at the location the photographer is standing (e.g., characteristic rocks or vegetation). By virtually capturing sites using DSMs anchored by DGPS ground‐control points, we consider that future surveyors may find it easier to relocate exact survey sites. The photogrammetric models created during this process can be georeferenced with precise DGPS and then accessed in the field using a standard smartphone or tablet. Because DSMs are high‐resolution and can be viewed from multiple angles in the field, they represent an improvement over 2D images. In effect, photogrammetry allows for the creation and marking of virtual permanent plots with the use of readily available photographic equipment.

The photogrammetric approach does have limitations, and we do not suggest that it should completely replace more conventional field‐based monitoring techniques. One drawback is cost; even though this method is less expensive than using UAVs, photogrammetric software can be expensive (an educational license for Agisoft Metashape costs ~$549 USD), in addition to the cost of a smartphone. Furthermore, while field time is reduced, compiling photos into a DSM can be time‐consuming (taking hours for a single model or days for many models, depending on computational capacity; however, cloud‐based processing can speed up this process). Partnerships between conservation agencies and universities could alleviate some of these constraints. Under such a model, agency staff or volunteers could be responsible for taking field photos with personal smartphones or cameras and university students could take on the work of creating photogrammetric models and even taking measurements as part of a project‐based or service learning class. Open‐source options for photogrammetric software are becoming more common, while educational licenses for image processing software are often less costly than traditional licenses. Such partners should take steps to facilitate long‐term storage of photos, DSMs, and any measurements or identifications in data repositories with detailed metadata.

As alpine and other rare plant communities face increasing challenges due to global change and a growing interest from hikers and other alpine visitors, there remains a pressing need for effective, reproducible, and affordable methods for long‐term monitoring. This photogrammetric approach effectively minimizes the time and resources spent in fragile ecosystems (and in harsh conditions), while maximizing the potential for data collection and accessibility of both data and field participation. As such, we believe this approach is well‐suited to ecosystems like those of the alpine zones of the northeastern United States, which are at‐risk, culturally important, and heavily trafficked, while also being managed by a diversity of agencies (including nonprofits and state and federal governments) with unique usage and research guidelines. This approach could also be useful in other systems where sensitive, sessile communities would benefit from long‐term monitoring, but where permanent plots may not be feasible, such as desert crusts, grasslands, polar or tundra systems, and rock outcroppings. Future applications of photogrammetry could also involve the further development of trait measurements that could be recorded within photogrammetric models, such as leaf area or floral traits. We also hope to utilize this approach for incorporation in displays or interactive art exhibits at visitor centers, by 3D printing, painting, and displaying our models as examples of alpine plant community differences across space and time.

## AUTHOR CONTRIBUTIONS

J.L.G., A.J.T., and A.E.P. conceived and designed the research in this manuscript. M.I.J.C. and A.J.T. completed the fieldwork and data collection. A.J.T., J.L.G., A.E.P., and M.I.J.C. drafted the manuscript text and contributed revisions. All authors approved the final version of the manuscript.

## Supporting information


**Video S1**. A video depicting a virtual permanent plot created from photos taking on Mt. Guyot, New Hampshire, USA. This video demonstrates how DSMs can be positioned and viewed within Agisoft Metashape software.

## Data Availability

3D image models created during this project can be found online on FigShare (https://figshare.com/s/03a500bf7717afe3a9a6).
